# GIS-Based and Outranking Approach to Assess Suitable Pig Farming Areas in the Mediterranean Region: Valencian Community

**DOI:** 10.3390/ani11041151

**Published:** 2021-04-17

**Authors:** Consuelo Calafat-Marzal, Áurea Gallego-Salguero, Marina Segura, Salvador Calvet-Sanz

**Affiliations:** 1 Departamento de Economía y Ciencias Sociales, Universitat Politècnica de València, 46022 Valencia, Spain; 2 Departamento de Ingeniería Cartográfica, Geodesia y Fotogrametría, Universitat Politècnica de València, 46022 València, Spain; augalsal@cgf.upv.es; 3 Departamento de Economía Financiera y Actuarial y Estadística, Universidad Complutense de Madrid, 28223 Madrid, Spain; marina.segura@ucm.es; 4 Instituto de Ciencia y Tecnología Animal, Universitat Politècnica de València, 46022 València, Spain; salcalsa@upvnet.upv.es

**Keywords:** depopulation, PROMETHEE, AHP, swine sector, livestock farming, small farms, medium farms

## Abstract

**Simple Summary:**

The livestock sector is the main source of income for the inhabitants of many rural areas, but the environmental problems generated by this industry have led to the emergence of new regulations to make the sector more environmentally friendly. The aim was to identify suitable areas where the livestock sector could be developed, complying with all sectoral standards, and classify the outranking suitable municipalities according to socio-economic, sectoral, and environmental factors. Socio-economic, to make it a key sector for the development of rural areas with depopulation problems; sectoral, to avoid generating health risk; and environmental, to avoid risks of water pollution and degradation of natural and forest areas. The results showed that there was a clear correspondence between the most suitable areas for pig farming and areas with depopulation problems or where rural tourism had not been developed. This information allows the design of public management instruments to prioritise the development of the sector in these areas.

**Abstract:**

The concentration of livestock production is problematic due to environmental concerns. European regulations are guiding the sector to become increasingly sustainable and, at the same time, maintaining the population in rural areas. The aim was to determine suitable areas in municipalities where livestock is presented as a market option. The methodology applied was based on the combination of multi-criteria methods and geographic information system (GIS) techniques, following three steps: removal of unsuitable zones by sectoral regulations (STEP 1); removal of unsuitable zones due to urban planning, and environmental recommendations (STEP 2); and evaluating the resulting areas depending on the importance of socio-economic, sectoral, and environmental characteristics. This study was based in a Spanish region with ongoing conflicts over land use on the coast but with a high number of rural municipalities at risk of depopulation in the interior. The results showed that 33% of the municipalities of the Valencian Community (VC) had suitable and outranking areas for the development of the swine sector. The 43 municipalities with the highest scores were because of the socio-economic factor and confirmed that suitable livestock development in municipalities with the highest risk of depopulation and low rural tourism activity was a key issue for development.

## 1. Introduction

The swine sector is the first livestock production industry in terms of economic importance in Spain. In 2019, it accounted for around 14% of the final agricultural production and 39% of the final livestock production, with significant growth in production, census, and the number of farms [1] due to the thrust of foreign markets and the competitiveness of the sector in the world market.

Worldwide, the EU-28 is the second-largest producer of pork after China. Individually, Spain is the fourth-largest producer (after China, the USA, and Germany), while in Europe, Spain is the second-largest producer after Germany, with 19% of the tonnage produced [2] and increasing exports to third countries, especially China and other Southeast Asian countries. In addition, it is the first EU country in the number of animals, with about 21% of the EU census [1,2].

Intensive livestock production entails several environmental risks related to the concentrated production of manure [3,4,5,6,7]. Intensive swine production produces large amounts of liquid manure (slurry), which produces ammonia emissions [8,9,10] and, if applied to land at excessive rates or in improper conditions, they contribute to groundwater pollution with nitrates [11,12]. Land planning contributes to more rational distribution of animal production and manure disposal [13], and it is, therefore, essential to reduce the environmental risks related to intensive livestock production [5].

Social concerns also arise when considering intensive animal production [6,8,14]. The production is controlled by a small number of large companies (vertical integration), which involves a loss of decision potential and income for farmers. The uniform production systems promoted by the vertical integration of systems also involve a loss of local genetic resources. Intensive livestock production uses large amounts of feeds elaborated with ingredients from global markets (for example, cereals and soybean meal) and is, therefore, unrelated to local agriculture in terms of crops but not in terms of manure management [15]. On the contrary, it depends on the local territory in the consumption of other resources such as water.

Intensification leads to a more automated production system, which can limit the social and economic impact in rural areas [16]. However, there are mixed livestock and crop production systems in which animal production plays an important role in terms of economic sustainability [5]. In these cases, the use of livestock manure provides an excellent opportunity of providing high-value fertilisers at low cost [13]. If the livestock production is properly integrated into the socio-economic context of a rural area, it can contribute to maintaining the population [17].

The new European and national regulations oriented towards a more sustainable economy regulate new scenarios for the livestock sector, such as the European Green Deal and Royal Decree 306/2020. The farm to fork strategy, as an action of the European Green Deal, aims to strengthen the sustainability of food systems. In this respect, livestock must be increasingly sustainable and, at the same time, be able to be a profitable and income-generating activity.

In Spain, the basic rules for the management of intensive pig farms were published in 2020 (RD 306/2020) to define a development strategy for the coming decades, which will position it in world markets, reduce the risks associated with internationalisation, and integrate the social and environmental challenges demanded by society. The evaluation of the territorial suitability for the development of sustainable livestock (minimising environmental risks) is key to increase the potential of the sector to contribute to income generation (social aspects) and allow sectoral development.

Studies to identify suitable areas for the location of intensive pig farms have been of interest in many countries, particularly in China [3,4,18,19] and in Europe [20,21,22]. The methodology used in several studies combined geographical information systems (GIS) and multiple-criteria decision-making (MCDM) methods [3,4,23,24,25,26,27].

The study was based on a Spanish region located on the Mediterranean coast: the Valencian Community (VC). In this region, there are ongoing conflicts over the land use on the coast, but with a high number of rural municipalities at risk of depopulation in the interior and where livestock farming is emerging as an option for generating income that would allow the population to settle in these rural areas, with the swine sector being the main alternative in terms of turnover.

The aim of this study was to evaluate the territory of the VC to identify municipalities with socio-economic, sectoral, and environmental outranking characteristics, in which there are suitable surface areas for the location of pig farms. The starting hypothesis was that the less economically developed the inland zones, the most suitable for the location of farms they would be and could serve as a driving force for the revitalisation of these areas.

The novel contribution of this study was the definition and validation of a methodology based, as a first step, on the elimination of territorial areas by legal regulations or environmental recommendations and, as a subsequent step, used multi-criteria analysis techniques for analysing the importance of socio-economic indices (which included indicators of depopulation, the evolution of the active population, affiliation by sector of activity, and the development of tourism), environmental variables (protected natural areas, percentage of forest area, and degree of vulnerability to groundwater pollution), and sectoral variables (swine density, livestock density, percentage of common undeveloped land available, and types of crops) to identify suitable livestock areas.

## 2. Materials and Methods

### 2.1. Study Area

**Figure 1:**
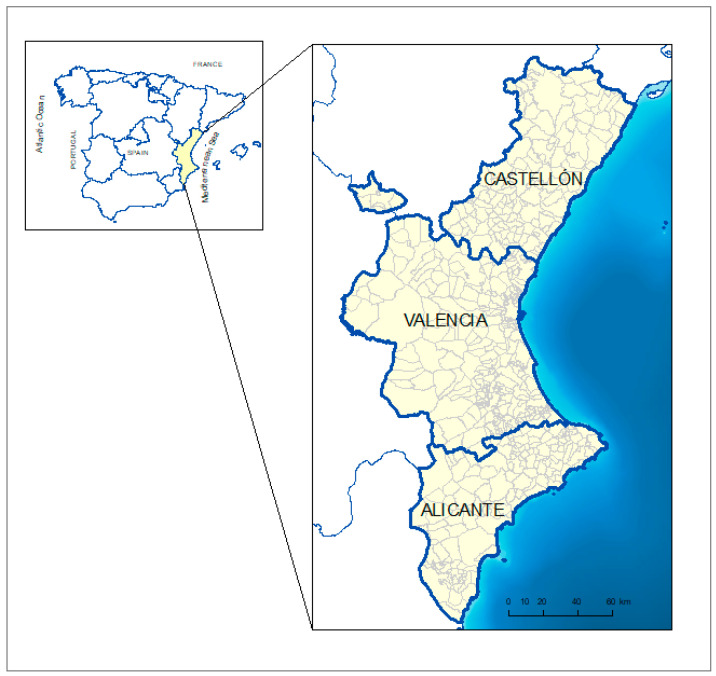
Valencian Community location map.

The study area (Figure 1) was the entire territory of VC, a region located in eastern Spain, which is part of the West Mediterranean area of Europe, covering 23,255 km^2^. Nearly 5 million inhabitants live in this area, and the agri-food sector accounts for approximately 12% of the Gross Domestic Product (GDP). Approximately 44% of the land area is used for agricultural purposes. The topography delimits regional environments that differ substantially, establishing three orographic areas: inland, intermediate, and coastal zones. The inland zone is characterised by pasture, forest, scrub, thicket, towns, extensive wood production, land abandonment, and terraces. The intermediate zone includes towns, dryland uses, urbanisation, and irrigated crops. There are conflicts regarding the land use in the coastal zone next to the Mediterranean Sea, where substantial industry and tourism occur [28].

According to the Valencian agricultural sector report for 2019, animal production in 2019 represented 22.6% of the final agricultural production in VC, and livestock production accounted for 77.4% of animal production. The swine sector represented 54% of livestock production, indicating that it was the most important sector in terms of volume of production. The value of livestock production in the last five years (2015–2019) has increased by 17.5%, while the value of swine production has increased by 47%, with a production value of 313,587 million euros in 2019.

In the VC, there were 925 pig farms, 60% of which were in the province of Castellón and 36.2% in Valencia, and a few in Alicante (Table 1). Pig farms for pork production might house sows, piglets, and fattening pigs. From these farms, the following types of farms were distinguished:

- Farms with reduced capacity—Farms with a capacity of up to 5.1 livestock units (LU).
- Group 1—Farms with a capacity of up to 120 LU.
- Group 2—Farms with a capacity of more than 120 LUs and up to 480 LUs.
- Group 3—Farms with a capacity of more than 480 LUs and up to 720 LUs.

In addition, there were pig farms that were not dedicated to the production and fattening, called extended distance farms, engaged in selection, multiplication, rebreeding of breeding stock, the transition of nulliparous breeders, pig semen collection centres, and quarantine farms.

Group 2 included most of the farms and LUs (51% and 66%, respectively). Group 1 represented 40% of the farms but only 19% of the LUs. Group 3 represented 3% of the farms and 12% of the LUs.

### 2.2. Data Sets

The farms were georeferenced with centimetric accuracy, with a GNSS absolute positioning receiver. The locations obtained were referred to the Universal mapping projection of Universal Transverse Mercator (UTM), zone 30, and the reference Geodetic System European Terrestrial Reference System 1989 (ETRS89).

**Figure 2:**
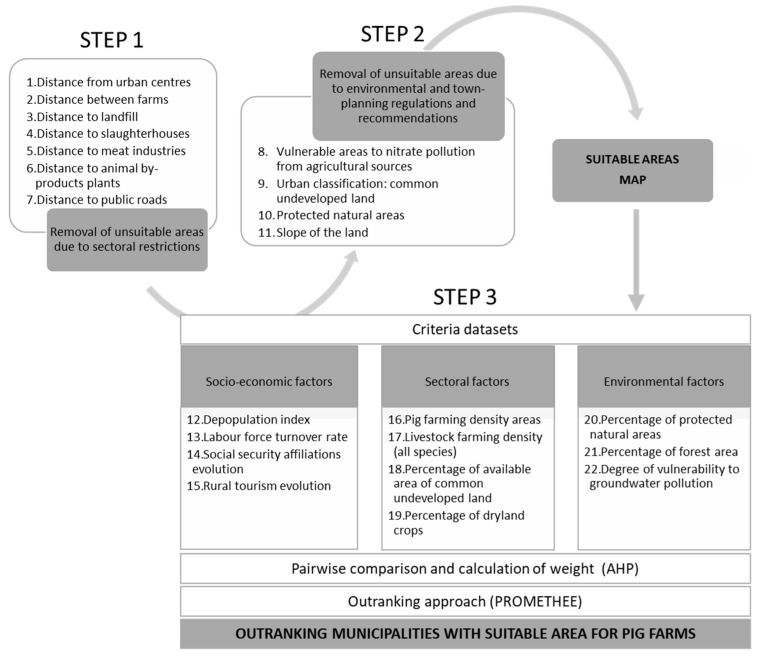
Flowchart of the land use suitability analysis for pig development.

The flowchart (Figure 2) of the GIS-based and multi-criteria analysis we used shows three steps. Based mainly on GIS modelling, it consisted primarily of two steps: the elimination of the areas not suitable because of legal regulations and other sectoral and environmental restrictions and/or recommendations.

The elimination of areas not suitable because of restrictions of legal regulations was mainly based on compliance with the basic standards for intensive pig farming (RD 306/2020): minimum distances between farms and between farms and other establishments or facilities (landfills, slaughterhouses, animal by-products not intended for human consumption plants, and roads). Other restrictions, which are not specified in the previous regulations, were the municipal regulations that indicated the location of livestock farms in the urban development classification of common non-developable land, outside protected natural areas, and, furthermore, according to Directive 91/676/EEC (and its national and regional transposition in RD 261/96 and Decree 11/2004, respectively) vulnerability to nitrate pollution in groundwater and the capacity of the soil to assimilate these was taken into account in order to minimise the risks of nitrate pollution. In addition, it was recommended that farms should not be located in areas with high slopes.

To determine the areas suitable for the location of livestock farms, restrictive criteria were selected based mainly on compliance, with the basic regulation for intensive pig farms (RD 306/2020), which established the minimum distances between the farms, and between the farms and other establishments or facilities (STEP 1), and the environmental and town-planning recommendations (STEP 2).

Once the areas suitable for the installation of pig farms were identified, the most appropriate areas were determined, in which livestock farming was outlined as an option for economic growth (STEP 3), considering socio-economic, sectoral, and environmental factors.

Table 2 describes the sources and formats of the variables considered.

#### 2.2.1. Description of STEP 1 Variables

In this step, the areas not suitable due to regulations for intensive pig farms were removed. The following criteria describe the minimum distances to be considered from the centre of a pig farm to the following locations:

- Criterion 1 (C_1_)—Distance to urban centres (km): minimum distance of 1 km for farms of all sizes.
- Criterion 2 (C_2_)—Distance between pig farms. For this purpose, the influence area was calculated of pig farms with sufficient production capacity to be used for the market (921 pig farms) depending on the size of the farms (Table 3). Further eliminated were the farms for self-consumption, where the animals were raised exclusively for family consumption, with a maximum production per year of 3 fattening pigs and without the availability of breeding stock.
- Criterion 3 (C_3_)—Distance to authorised landfill sites. This will depend on the size of the farm (Table 3).
- Criterion 4 (C_4_)—Distance to slaughterhouses: minimum distance of 2 km for farms of all sizes.
- Criterion 5 (C_5_)—Distance to meat industries: minimum distance of 500 m for all sizes of farms.
- Criterion 6 (C_6_)—Distances to registered establishments or plants for the treatment of animal by-products not intended for human consumption (ABPs) and products derived thereof (animal by-products regulation) of category 1 and 2 with the treatment of carcasses (Regulation (EC) No 1069/2009 of the European Parliament and of the Council). The minimum distances considered depended on the size of the holdings and were included in Table 3.
- Criterion 7 (C_7_)—Distance to public roads: Minimum distance of 100 m to railways, highways, motorways, and roads of the National Network.

For this study, only groups 2 and 3 farms (small/medium scale) were considered and, in consequence, their limitations. In Spain, the trend is towards large farms. To assess locating an extended distance farm, a more detailed study should be carried out, as the distances were more restrictive in some criteria.

#### 2.2.2. Description of STEP 2 Variables

In this step, the areas unsuitable because of environmental and town-planning regulations and recommendations were removed.

- Criterion 8 (C_8_)—Nitrate-vulnerable zones caused by nitrates from agricultural sources.

The designation of nitrate-vulnerable zones was established to designate those territorial areas whose runoff or seepage affected or might affect water bodies that were polluted by nitrates or at risk of being polluted. The regulations governing these zones are: Directive 91/676/EEC; Royal Decree 261/1996, Order 10/2018, and Decree 86/2018.

The areas classified as vulnerable did not imply the prohibition of farming but rather the prevention of nitrates from agricultural sources polluting ground and surface waters by promoting the use of good agricultural practices, especially in the application of manure. Therefore, to select the municipalities with the least restrictions for manure application, municipalities vulnerable to nitrate pollution were removed.

- Criterion 9 (C_9_)—Local zoning laws.

Law 5/2014, of 9 December, by the Valencian government on non-developable land states that livestock farms were included in the urban development classification of common non-developable land. All other urban development qualifications were removed.

- Criterion 10 (C_10_)—Protected natural areas.

Natura 2000 is a European ecological network of biodiversity conservation areas, consisting of Special Areas of Conservation (SAC) designated by the member states under the Habitats Directive (92/43/EEC) and Special Protection Areas (SPAs) designated under the Birds Directive (79/409/EEC, amended by Directive 2009/147/EC).

It removed wetlands and their areas of influence, SACs, SPAs, buffer zones for natural monuments, protected landscapes, municipal nature sites, and sites of community interest (SCIs).

- Criterion 11 (C_11_)—Slope of the land

The slope of the land for livestock had to be less than 25% [3,4], so areas with a slope greater than 25% were eliminated using the digital model of the land in raster format of 25 m of pixels of the VC.

#### 2.2.3. Description of STEP 3 Variables

In this step, outranking selection of municipalities with areas suitable for pig farms 263 according to socio-economic, sectoral, and environmental characteristics is carried out with 264 a multi-criteria models. In this step, municipalities are prioritised, and not suitable areas, 265 because socio-economic, sectoral, and environmental characteristics are at the municipali-266 ties level not at area level. Then the suitable areas in the same municipalities would have 267 the same scores, since they have the same socio-economic, sectoral and environmental 268 characteristics.

The assessment of suitable municipalities for the location of pig farms will make it possible to classify the territory into areas where it can contribute to economic development. Using multi-criteria techniques, the importance of socio-economic, sectoral, or environmental aspects will be determined. For the evaluation of these variables, municipal data have been used and overlaid with the results of STEPs 1 and 2. In this way, municipalities that have available and suitable land for pig farms are analysed and municipalities that do not have suitable land are removed from the analysis. The municipalities are identified by their Municipal Code, which is made up of 5 digits; the first two digits to identify the province (03 for Alicante, 12 for Castellón and 46 for Valencia) and the following to identify the municipality. There are 542 municipalities in the VC (141 in Castellón, 134 in Castellón and 264 in Valencia).

##### Socio-Economic Factors

- Criterion 12 (C_12_)—Depopulation index.

To characterise the phenomenon of depopulation in the municipalities of the VC, a depopulation index was obtained from the indicators proposed in Decree 182/2018 of the Valencian Government. The data are from 1999–2019. Table 4 includes the indicators used, together with a description of these and the proposed thresholds.

Depending on the value of the demographic indicators, the municipalities could be classified according to the depopulation risk:

Very high risk—The municipality met the criteria for all the indices.

High risk—The criteria were met for five indicators.

Moderate risk—The criteria were met for four indicators or, without meeting these indicators, the population of the municipality was less than or equal to 120 inhabitants, or the population density was fewer than 12.5 inhabitants/km2.

In VC, there were 23 municipalities with very high risk, 27 with high risk, 95 with moderate risk, and 397 without risk.

- Criterion 13 (C_13_)—Labour force turnover rate.

This relates to the size of the groups of working-age starting their activity with those in which they are leaving the labour market. The aim was to measure the ability of a population to replace those who retire. The average annual rate of change between 2009 and 2019 was calculated.

Labour force turnover rate = Population aged 20–29 × 00/population aged 55–64(1)

If the average annual rate was greater than 100, it indicated that there was a renewal of the active population in that municipality, so a value of 0 was assigned. If the average annual rate was less than 100, i.e., there was no renewal of the active population, a value of 1 was assigned to that municipality.

- Criterion 14 (C_14_)—Social security affiliations evolution in the field of agriculture, livestock, forestry, and fisheries.

This was obtained in the number of social security affiliates in agriculture and the percentage it represented of the total affiliates in all activity sectors from 2012 to 2019. Municipalities with an average agricultural affiliation percentage higher than the provincial average were assigned a 1, municipalities with an average agricultural affiliation higher than 25% of the provincial average were assigned a 2, and those with an average agricultural affiliation higher than 50% were assigned a 3.

The agricultural affiliation average in the VC was 4.5% of the total affiliations; in the province of Alicante it was 4%, in Castellon 6.5%, and in Valencia 4.4%.

- Criterion 15(C_15_)—Rural tourism evolution.

Rural tourism is a market option in municipalities where the number of places in rural houses and hostels was increasing from 2009 to 2019. The increase in the number of farms could generate odour problems that curb rural tourism. The municipalities where rural tourism grows more than the provincial average were assigned a 0; the municipalities that increased, but less than the provincial average, were assigned a 1; and those that decreased were assigned a 2.

Rural tourism in the VC increased by 17.6%, with 36.8% in the province of Alicante and 30.3% in Valencia, while in the province of Castellón it decreased by 3.5%.

##### Sectoral Factors

- Criterion 16 (C_16_)—High pig farming density areas.

In municipalities with a high density of pig farms, the health and safety risks for pig farms were increased. Thus, the values assigned to the municipalities were as follows: zero for municipalities with a high density of pig farms; 1 for municipalities with low density; 2 for municipalities without pig farms or with less than 1 LU.

Municipalities with more than 2 LU/ha Utilised Agricultural Area (UAA) were considered high livestock density municipalities [29], and those with between 1 and 2 LU/ha of UAA were considered low livestock density municipalities.

- Criterion 17 (C_17_)—High livestock farming density (including all livestock species).

The areas of high livestock density were determined using the database of livestock farms in the VC, where both the species and the number of animals were available. The process followed for its determination was similar to that for pig farming density areas.

- Criterion 18 (C_18_)—Percentage of available areas of common undeveloped land.

The indicator distinguished municipalities depending on available agricultural areas. The percentage of common undeveloped land in each municipality was calculated with respect to the average values for the province. Municipalities with a higher percentage of available agricultural areas than the provincial average, more agrarian, were assigned value 1, and municipalities with percentages lower than the provincial average were assigned a value of 0.

The percentage of common undevelopable land in the VC was 32.1%, in Alicante 34.9%, in Castellón 29.1%, and in Valencia 32.2%.

- Criterion 19 (C_19_)—Percentage of dryland crops.

Dryland crops, in general, differ from irrigated crops in that they are less labour-intensive, less productive, and less profitable. It was considered that the combination of extensions of dryland crops with livestock farms increased diversification for the farmer and improved their income [17].

Municipalities with a percentage of non-irrigated crops above the provincial average were assigned a 1, and those with a higher percentage of the irrigated area were assigned a 0.

Rainfed crops in the VC represented 45.7% of the useful agricultural area, in Alicante 38.3%, in Castellón 63.9%, and in Valencia 40.7%.

##### Environmental Factors

- Criterion 20 (C_20_)—Percentage of protected natural reserves in relation to the total municipal surface area.

Indicates the degree of landscape importance of the municipality, considering the percentage of protected natural reserves in Natura 2000 Network, and in accordance with Law 42/2007 on Natural Heritage and Biodiversity.

Municipalities with a percentage of protected areas above the provincial average were assigned the value 1, and municipalities below were assigned the value 0.

The percentage of protected areas in the VC represented 31.5%, in Alicante 26.6%, in Castellón 46.9%, and in Valencia 26.4%.

- Criterion 21 (C_21_)—Percentage of forest area in relation to the total municipal surface area.

This indicator describes the characteristics of municipal land occupation. In the VC, the municipalities with the largest areas of forest were related to municipalities with fewer possibilities for the development of economic activities. It was therefore considered that livestock farming could be an economic boost for these municipalities. Municipalities with a percentage of forest areas above the provincial average were assigned value 1, and municipalities below were assigned value 0.

The percentage of forest areas in the VC represented 46.85%, in Alicante 42.69%, in Castellón 63.4%, and in Valencia 34.47%.

- Criterion 22 (C_22_)—Degree of vulnerability to groundwater pollution.

This criterion refers to the risk of groundwater deterioration due to potentially polluting discharges or activities. The cartography on the vulnerability of groundwater in the VC provided for the division of the territory into areas with different degrees of pollution risks. These areas were characterised by the degree of groundwater protection according to the quality and availability of water resources, which made it possible to determine the suitability and limitations of the territory for livestock use.

Five categories (very high, high, high, medium, low, and very low) were established for the sensitivity to pollution of aquifers depending on the soil permeability, unsaturated zone thickness, and groundwater quality.

For each municipality, the most representative category was obtained, and municipalities, where the most representative vulnerability category was high or very high, groundwater pollution was assigned value 0; municipalities, where the most representative vulnerability category was medium, were assigned value 1; and municipalities, where the most representative vulnerability was low or very low, were assigned value 2.

### 2.3. Methods

Geographical information systems (GIS) and multiple-criteria decision-making (MCDM) are two tools that have been applied jointly for many purposes.

#### 2.3.1. GIS Methodology and Tools

**Figure 3:**
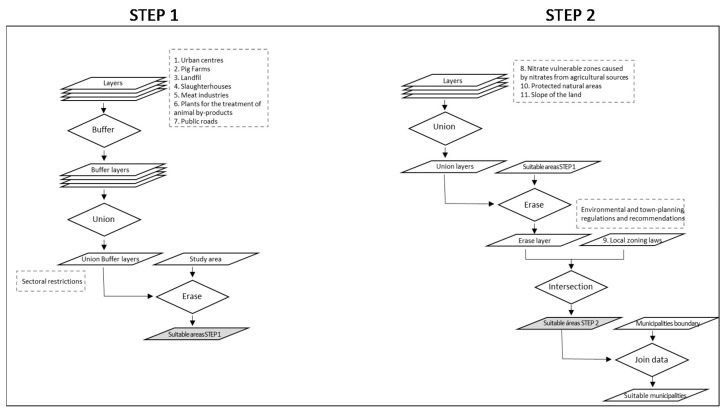
Geographical information (GIS) flowchart followed in STEP 1 and 2.

The GIS methodology and the tools used in STEP 1 and 2 are detailed in Figure 3. In these steps, areas suitable for pig farm locations were identified using GIS. These areas were obtained from the geographical layers of the study area. ArcGIS was the software used for the processing of spatial information.

In STEP 1, from the initial layers of the criteria described in Table 2 and Figure 2, the buffer was generated. The layers obtained from each criterion were overlaid to create a single layer containing the restrictions of the regulations for intensive pig farms. The areas of this layer were excluded from the study area to obtain a layer with the suitable areas from STEP 1.

In STEP 2, the layers of the criteria of this step (Table 2 and Figure 2) were overlaid and the layer obtained was removed from the layer resulting from step 1. The resulting layer was intersected with the local zoning law layer, from which the common undevelopable land was previously selected. Subsequently, areas smaller than 1000 m^2^ were removed, as they were considered insufficient to include a group 1 or 2 pig farm. The result was the STEP 2 suitable areas.

**Figure 4:**
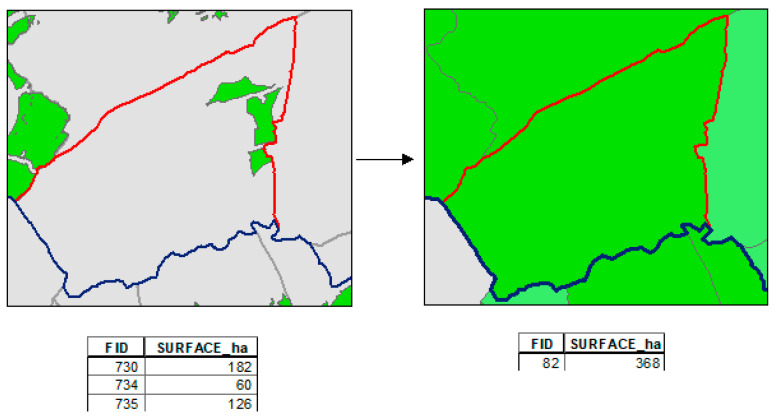
Example of change of observation unit from STEP 2 to STEP 3.

Then, with the layer of suitable areas from STEP 2, a spatial union of data was made with the layer of municipalities. The total surface of the suitable areas in each municipality was joined to the attribute table, changing the observation units to obtain the layer with the suitable municipalities that were considered in STEP 3 and in the multi-criteria evaluation (Figure 4).

**Figure 5:**
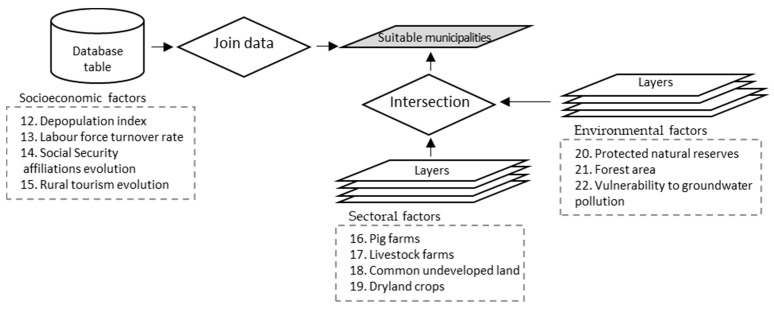
GIS flowchart followed in STEP 3.

In STEP 3, the variables used were municipal characteristics, so the units of observation referred to municipal variables. The GIS flowchart in STEP 3 was included in Figure 5.

In this step, the values of the socio-economic, sectoral, and environmental factors were associated with the municipalities with suitable areas for pig farms. First, the database containing the socio-economic factors was spatially joined with the attribute table of the municipalities with the suitable area layer. Secondly, the layers of the sectoral and environmental factors were intersected with the layer of the municipalities with suitable areas. Thus, the attribute table of the municipalities with suitable areas was completed with the criteria to be considered in the multi-criteria evaluation.

#### 2.3.2. MCDM Techniques

MCDM techniques are useful for selecting, classifying, and prioritising alternatives considering numerous attributes or criteria. In collaborative decision-making and policymaking, not only should multiple-criteria methods consider judgements or preferences of the different decision maker/s, but it also is very important that the method used would be able to make their subjectivity explicit [30].

The MCDM approach coupled with GIS was used to analyse multiple constraints that affect the siting of wind farms and solar farms [31]. Furthermore, recently, both tools together were used to select sanitary landfills in India, allowing municipal authorities to develop planning protocols for the near future [4,32]. Developed evaluation models to create the land suitability map integrated the analytic hierarchy process (AHP) method and GIS techniques. Other problems solved with multi-criteria techniques could be cited, such as their integration into decision support systems (DSS) for forest management, where this approach was useful. Different MCDM methods have been incorporated in ArcGIS [33], where AHP stood out by the number of uses. AHP and Preference Ranking Organisation Method for Enrichment Evaluation (PROMETHEE) was also integrated into DSS, for example, the LANdscape-scale succession and DISturbance (LANDIS) managed public forests in the USA, taking into account ecosystem services [34].

[35] (1986) initially developed PROMETHEE, an outranking method, to rank projects. This method was applied to solve other types of complex multi-criteria problems, such as production systems [36]. The non-compensatory nature of this method made it more appropriate for evaluating suitable livestock areas than compensatory multi-criteria approaches, as some factors cannot compensate others. Although unsuitable areas were removed in the first steps, for example, the risk increase for health due to higher livestock farming density in an area should not be compensated completely with positive performance in other criteria. To assess suitable livestock areas, this paper proposed a hybrid procedure based on two multiple-criteria techniques: PROMETHEE and AHP. The AHP method was used to determine the weights of criteria to be applied in PROMETHEE.

#### 2.3.3. Determining New Indicators for Municipalities Selection Depending on Socio-Economic, Sectoral, and Environmental Factors

**Figure 6:**
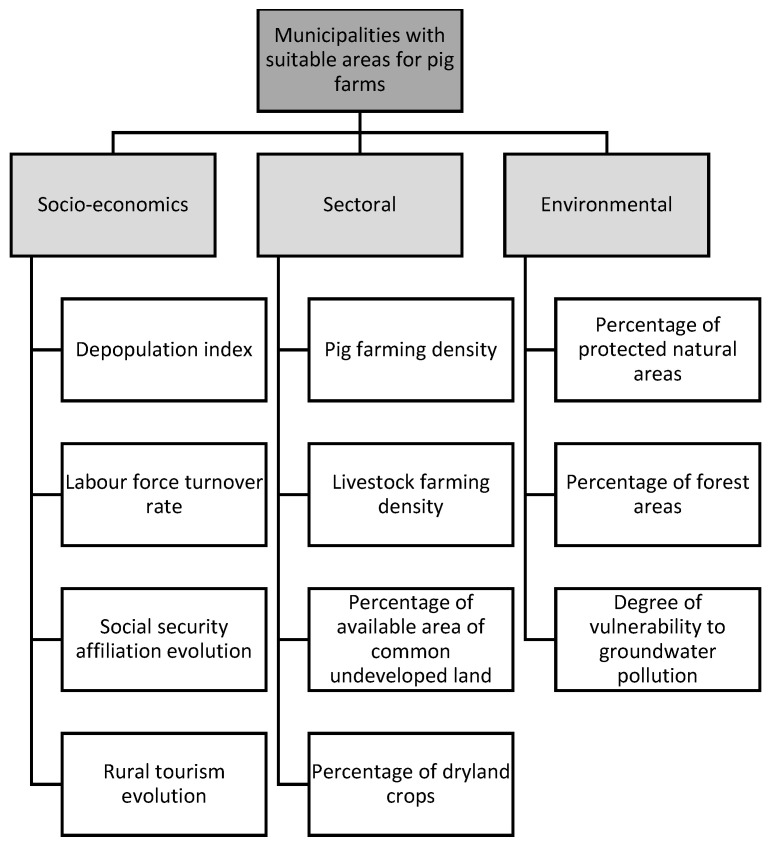
Hierarchy of criteria for evaluation of municipalities with suitable livestock area.

Once the criteria to perform the assessment of municipalities with suitable livestock areas were identified, they were structured as a criteria hierarchy (Figure 6).

The model proposed to select and rank the municipalities according to their suitability was based on PROMETHEE, in which the weights of criteria were elicited from a group of experts by the AHP method. The AHP application was carried out through a survey among experts from various areas related to livestock production management. Particularly, these experts included technical personnel from OCAPA (Regional Office of Agriculture, Fisheries, and Food) and experts from the College of Agronomists, animal science, veterinarians, environmental science, and regional authorities. In the survey, the experts were asked to indicate the relative importance between the criteria by pairwise comparison with respect to the criterion of the upper level in the hierarchy (Figure 3), using the Saaty scale. AHP initially sought input from individual experts rather than assuming a consensus to facilitate a compromise. The aggregated weights for five experts could then be obtained by using the geometric mean, so the consistency of the consensus matrices could be assured. The inconsistency of the individual matrices ranged from 0 to 0.06. Complete information on this method can be found in [37].

PROMETHEE considered the differences in performance of the municipalities and deleted the scale effect when the criteria were not measured in the same units. This method provided information about the conflicts between the criteria and allowed for sensitivity analyses to see the impact of the weights on the solution. For practical purposes, PROMETHEE II was applied in this work because it provided a complete ranking of municipalities in terms of knowing which were the best places to establish new livestock farms.

The last step of the methodology consisted of applying an outranking-based method as a tool to generate new indicators to assess functions of ESS which were obtained from individual indicators and grouped into a single index.

Table 5 represents the evaluation table for 178 municipalities with suitable areas, which were assessed by the selected criteria. We were interested in maximising some of them and minimising others.

The PROMETHEE method required information on the weights of the relative importance of the criteria; these weights were called w_1_, w_2_, …, w_k_, obtained in a previous step by AHP.

The PROMETHEE process for eliciting preferences was also based on pairwise comparisons such as AHP, although the former used a different nature. The comparisons depended on the difference between the evaluations of two areas on a particular criterion. Generally, the larger the difference between evaluations of the areas, the greater was the preference of the preferred areas, depending on the particular preference function of each criterion. In this study, due to the nature of the criteria, the preference had the maximum value of null, as the usual type of preference function was used. The preferences were represented by real numbers between 0 and 1.

The PROMETHEE method required information about the nature of the criteria in addition to their weights, which were the preference functions. The nature of the criteria and their evaluations established that the preference functions for all criteria were usual, which means ensuring that the area with better evaluation for a criterion had all the preferences. The objective of all criteria was to maximise, with the exception of the percentage of protected natural areas and percentage of forest areas criteria, in which the aim was to minimise (Table 6).

When comparing two municipalities for criterion j, the preference function between municipality a and municipality *b*, *P_j_* (*a*, *b*), is a function that depends on the difference between the behaviour of both municipalities *g_j_* (*a*) and *g_j_* (*b*). For all criteria, the preference function is usually:

(2) $$P_{j}=F_{j}\left[g_{j}\left(a\right)-g_{j}\left(b\right)\right]=F_{j}\left[d_{j}\left(a,b\right)\right]$$

where $d_{j}\left(a,b\right)=g_{j}\left(a\right)-g_{j}\left(b\right)$ and $P_{j}\left(a,b\right)=0$ or $P_{j}\left(a,b\right)=0$.

If $P_{j}\left(a,b\right)=0$, then $P_{j}\left(b,a\right)=1$.

For example, when comparing two municipalities with respect to the depopulation index (C_12_), the usual function assigned a strict preference to the best option that means 1 as a value of the preference. In this case, it was the option with the higher value because the depopulation index should be maximised. Municipality 12,044 with the maximum value in this criterion (6) was always preferred to any other with a value lower than 6.

Aggregated preference indices for each pair of municipalities with livestock suitable areas from the evaluation table; the weights and preference functions of the criteria are as follows:

(3) $$\pi \left(a,b\right)=\overset{n}{\underset{j=1}{\sum }}P_{j}\left(a,b\right)w_{j}$$

(4) $$\pi \left(a,b\right)=\overset{n}{\underset{j=1}{\sum }}P_{j}\left(b,a\right)w_{j}$$

where *π*(*a*, *b*) expresses the degree to which a is preferred over b.

When the comparison of each municipalities to the others, (*n* − 1) positive and negative outranking flows are defined as follows:

(5) $$\varphi^{+}\left(a\right)=\frac{1}{n-1}\underset{x\epsilon A}{\sum }\pi \left(a,x\right)$$

(6) $$\varphi^{-}\left(a\right)=\frac{1}{n-1}\underset{x\epsilon A}{\sum }\pi \left(x,a\right)$$

Whereas positive outranking flow expressed how much a municipality outranked all the others, the negative outranking flow indicated how much a municipality was overcome by the others. The positive and negative flows were indicators of the strength and weakness of the alternatives, in this case, the municipalities.

The balance between positive and negative flows of the areas is the net flow.

(7) $$\varphi \left(a\right)=\varphi^{+}\left(a\right)+\varphi^{-}\left(a\right)$$

The values of net flows were between −1 and 1, and the sum of all of them was 0. The nearer net flow was to 1, the better it was than other municipalities, and when the net flow was close to 0, the worse it was than the others. Therefore, the net flow was an indicator of how suitable the municipality was, aggregating socio-economic, sectoral, and environmental factors. The software used was D-Sight (2017).

The graphical representation of the results obtained by PROMETHEE was very useful for decision-making. In particular, the GAIA plane is a powerful visualisation to help understand the multi-criteria problem. The GAIA plane provided a visual representation of the uni-criterion net flows matrix. GAIA used a principal component analysis (PCA) to find a plane that allowed a 2D visualisation of the alternatives, which were the municipalities, as well as the criteria. GAIA plane also included the priorities of decision-makers by projecting the weights vector, known as decision stick, which clearly indicated the best municipalities [38,39].

Finally, the net flows of the municipalities with the suitable area were implemented into the GIS in the attribute table associated with the layer of the municipality, so we were able to represent and symbolise the results on a map.

## 3. Results

**Figure 7:**
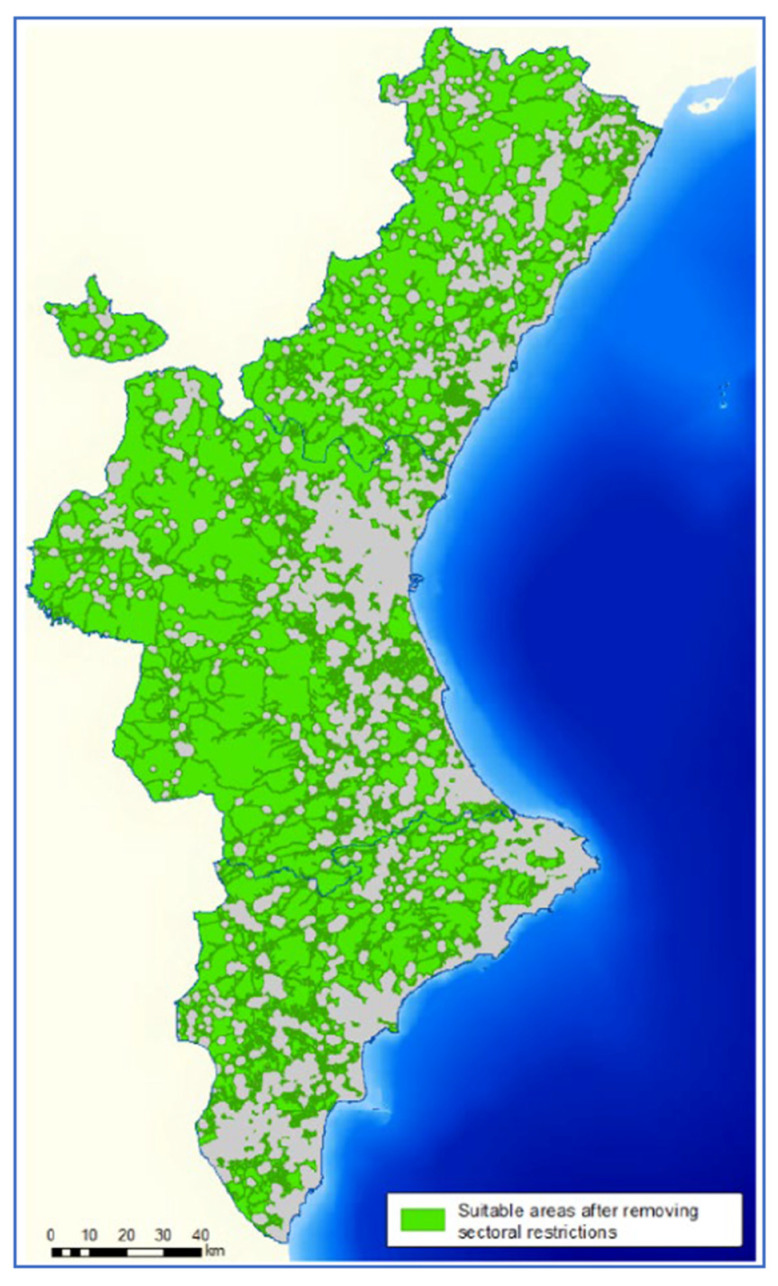
Suitable areas for siting pig farms in the VC in STEP 1.

The results obtained in STEP 1 determined the suitable areas according to the basic regulations for intensive pig farms (Figure 7). In this step, the area available for the location of pig farms was reduced to 56.4% of the total area of the VC.

**Figure 8:**
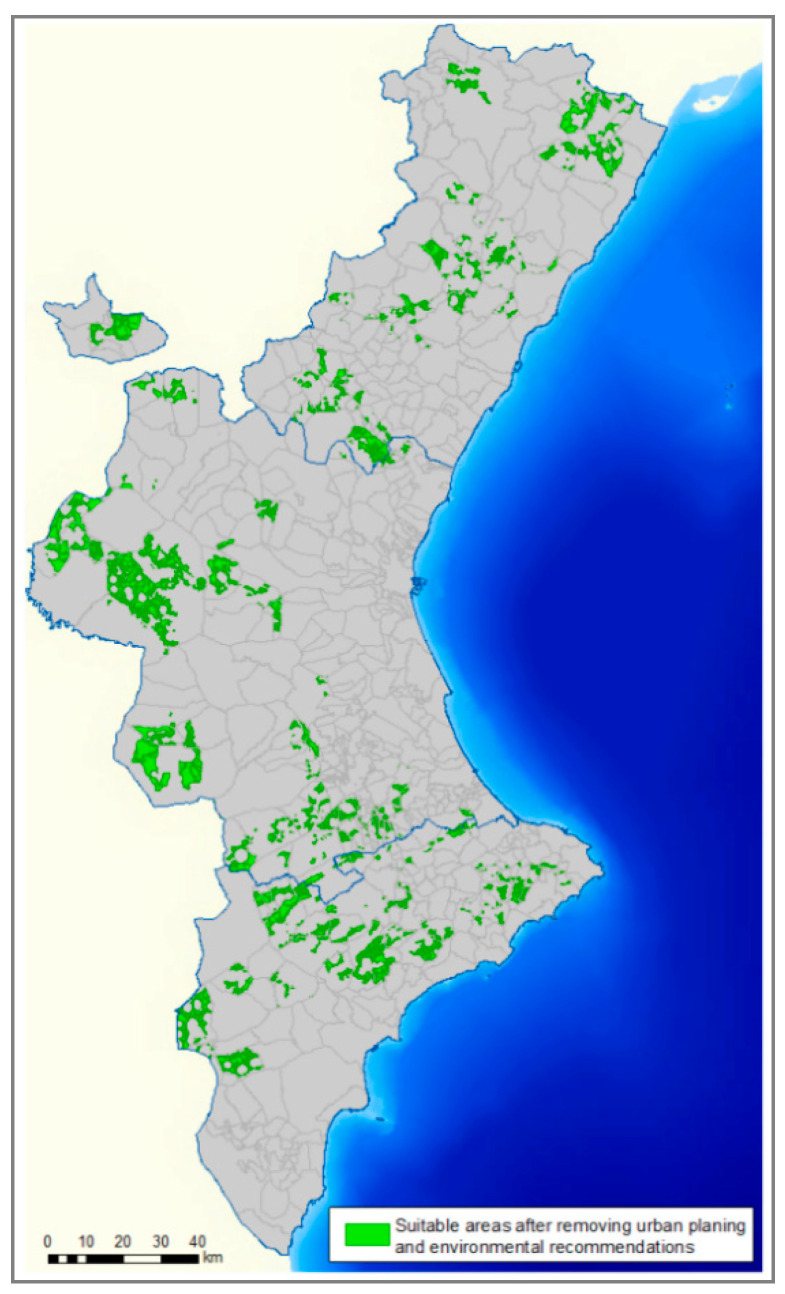
Suitable areas for siting pig farms in the VC in STEP 2.

The urban planning regulation and environmental recommendations for the siting of farms were considered in STEP 2. The area suitable for the siting of farms (Figure 8) was reduced to 4.9% of the total area. It showed that the suitable areas for the swine sector development were scarce and were in mountain or inland areas, mainly due to urban and environmental recommendations. The most suitable areas were in 178 of the 542 municipalities of the CV, which indicated that pig farms could be located in 33% of the municipalities. These municipalities were 56 in Alicante, 53 in Castellón, and 69 in Valencia.

In STEP 3, the areas resulting from STEP 2 were analysed to assess which municipalities were outranking depending on socio-economic, sectoral, and environmental factors by multi-criteria analysis. The combination of PROMETHEE, AHP, and GIS helped reduce the subjectivities, uncertainties, and hierarchical characteristics of the traditional land suitability assessment process.

The aggregated weights obtained by the AHP method are shown in Table 7. By factors, the most important factor was socio-economic, followed by the environmental criterion, and the least relatively important factor was sectoral. Among the socio-economic factor, the depopulation index was the principal criterion, followed by the evolution or rural tourism and labour force turnover rate with almost the same percentage, 25.5% and 23.5%, respectively. The percentage of protected natural areas appeared in the first position in the environmental factor; in the second position was the degree of vulnerability to groundwater pollution, and last was the percentage of forest areas.

**Figure 9:**
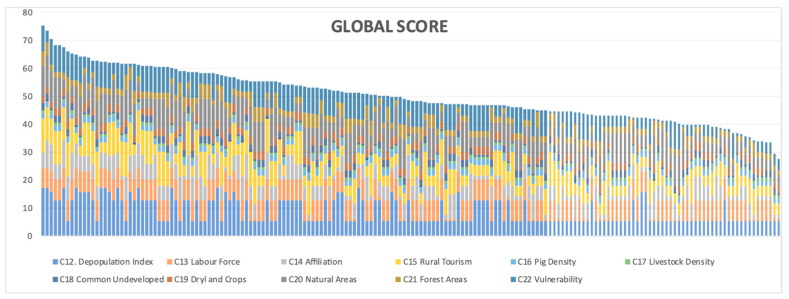
Score of 178 municipalities with suitable pig areas.

Figure 9 shows the aggregated score of all municipalities on a scale from 0 to 100. When net flow had a value of −1, the score in the figure was zero, and if it was +1, the score was 100. The different colours of the figure highlight the contribution of each criterion to the score. The maximum score of municipalities was 75.19. Generally, the score was the result of the most relevant criteria, i.e., depopulation index, labour force turnover rate, percentage of protected natural areas, and degree of vulnerability to groundwater pollution.

**Figure 10:**
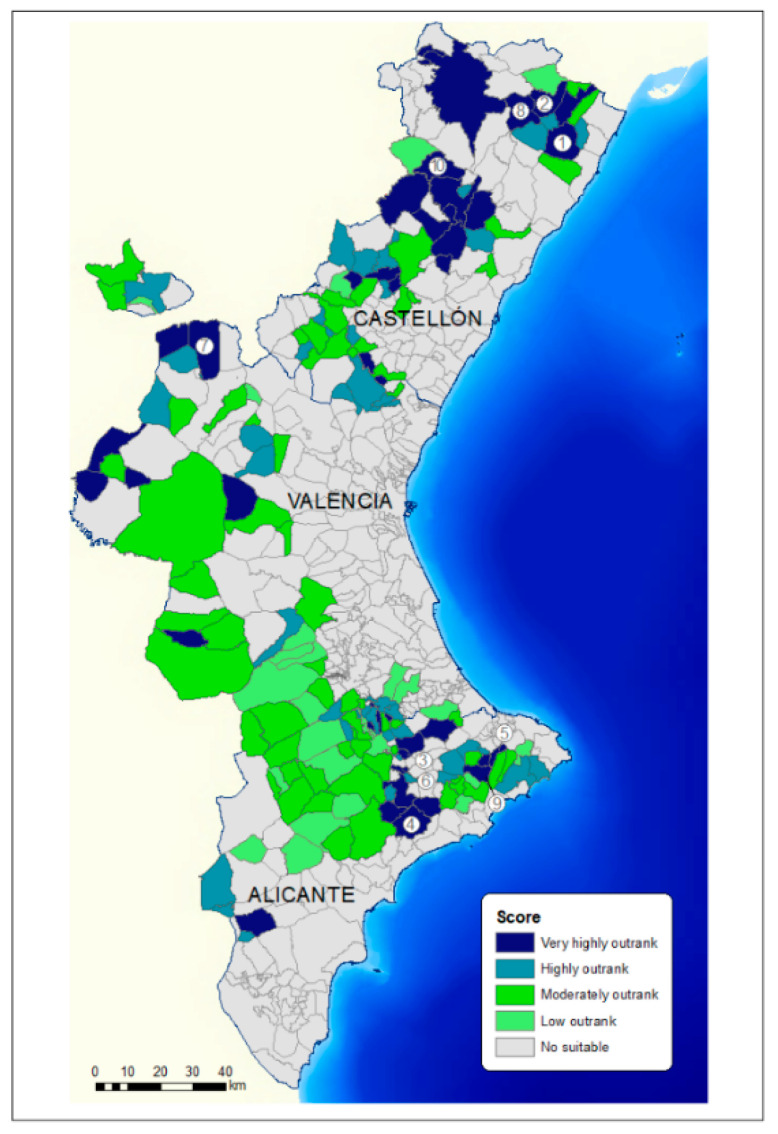
Map of the outranked municipalities depending on net flow value (the higher the score is the better the municipality).

Table 8 presents the specific classification criterion for evaluating the municipalities for swine sector development. The results of the net flow of the municipalities were implemented in GIS, obtaining the maps shown in Figure 8. In Castellón and Valencia, the municipalities with the best scores were in the inland areas, and in Alicante, they were more in the mountainous areas and closer to the coast. The municipalities with the highest scores (very high and high) were mainly located in Castellón (33 municipalities with very high and high) and the municipalities with medium levels (moderately and low) in Valencia and Alicante (42 in Valencia and 29 in Alicante). The first 10 municipalities are indicated in Figure 10, and there were 4 in Castellón, 5 in Alicante, and 1 in Valencia.

**Figure 11:**
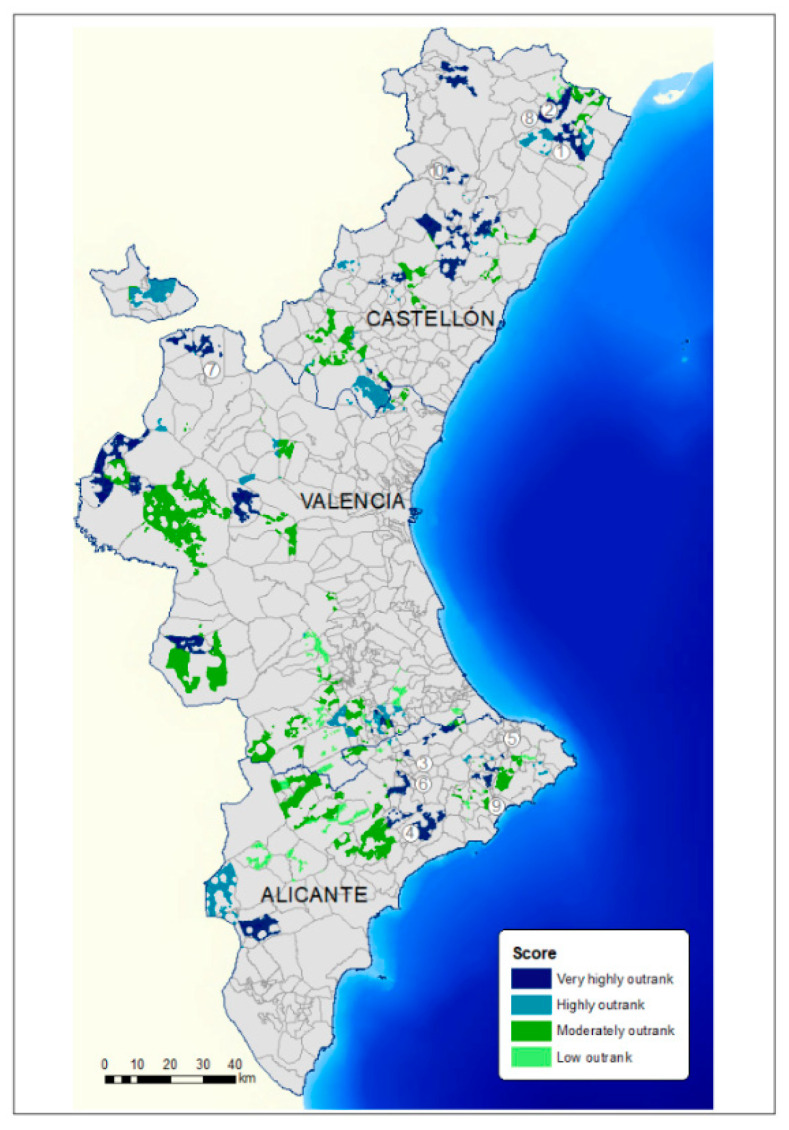
Map of the outranked areas in the suitable municipalities depending on the net flow value (the higher the score is the better the municipality).

Figure 11 shows the areas available in each of the municipalities. This showed which of the suitable areas were the best (very high score) and the worst (low score) for the location of pig farms.

**Figure 12:**
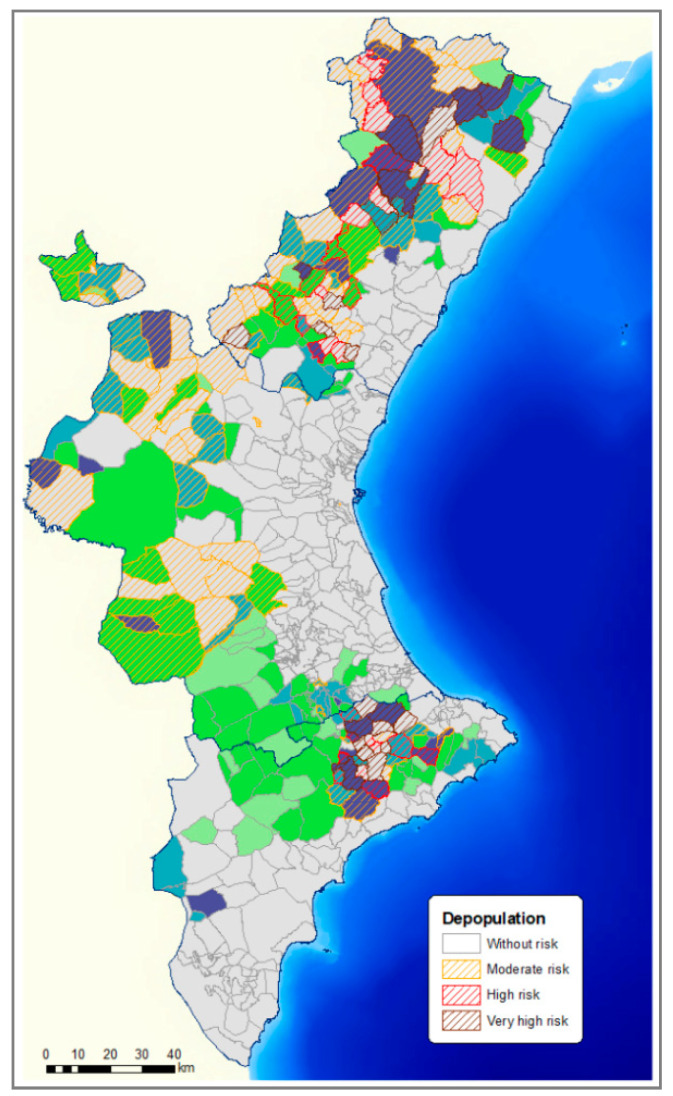
Relationship between municipalities with suitable land for swine sector development and municipalities with the risk of depopulation.

**Figure 13:**
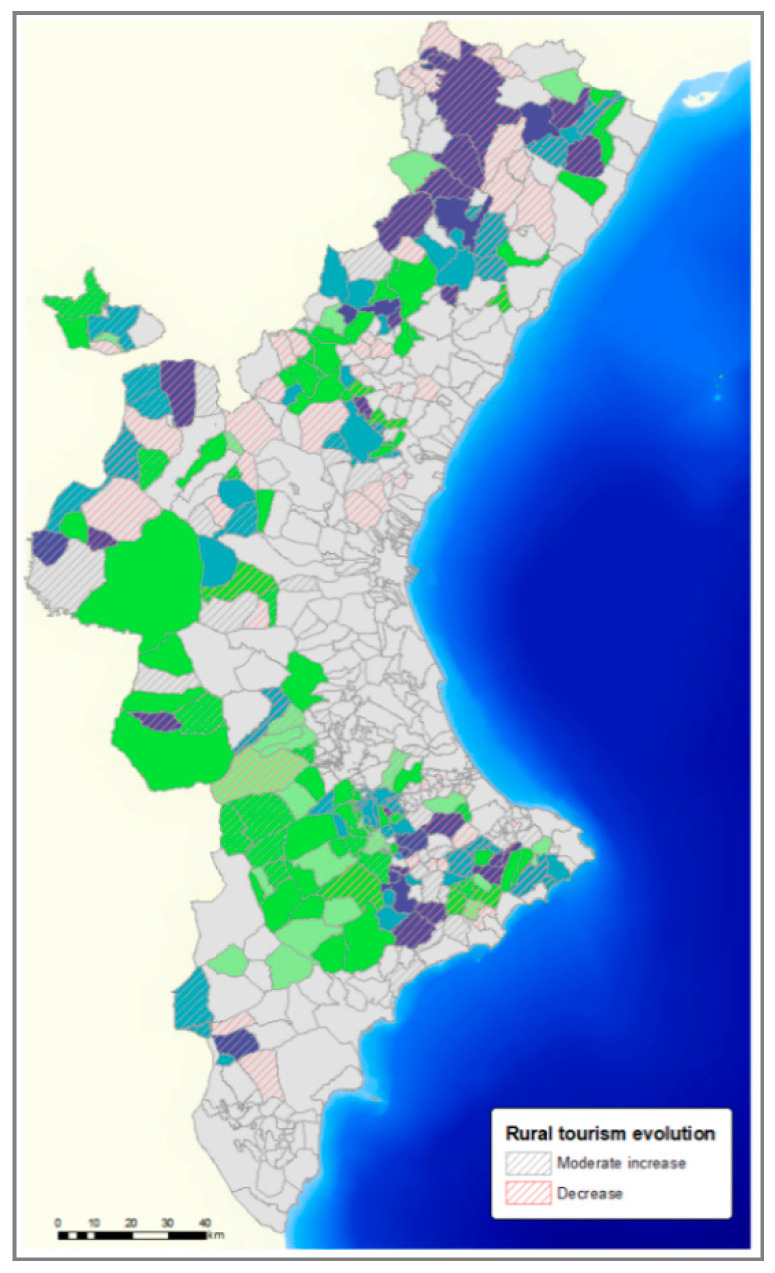
Relationship between municipalities with suitable land for swine sector development and municipalities with low evolution of rural tourism.

The 43 very highly outranked municipalities, according to the three factors, were mainly in the province of Castellón and Alicante (20 in Castellón, 13 in Alicante, and 10 in Valencia). In these municipalities, the best-scored was due to their socio-economic factor, and of these, due mainly to high depopulation risk and rural tourism evolution. In Figure 12 and Figure 13, the scores obtained with the depopulation risk (Figure 11) and rural tourism evolution (Figure 13) of each municipality were combined.

Some 48.97% of the municipalities at depopulation risk were municipalities with suitable land for pig farming (Figure 11), which indicated the strong relationship between the possibilities offered by this sector in depopulated rural areas.

The municipalities where rural tourism was only slightly developed or had been reduced accounted for less than 30% of the municipalities in the CV. There were 49.07% of these municipalities with suitable land for pig development (Figure 13).

In summary, the results obtained demonstrated that the swine sector could be a strategic industry in half of the municipalities at risk of depopulation and in the municipalities where rural tourism did not increase to the same extent as in the rest of the CV.

**Figure 14:**
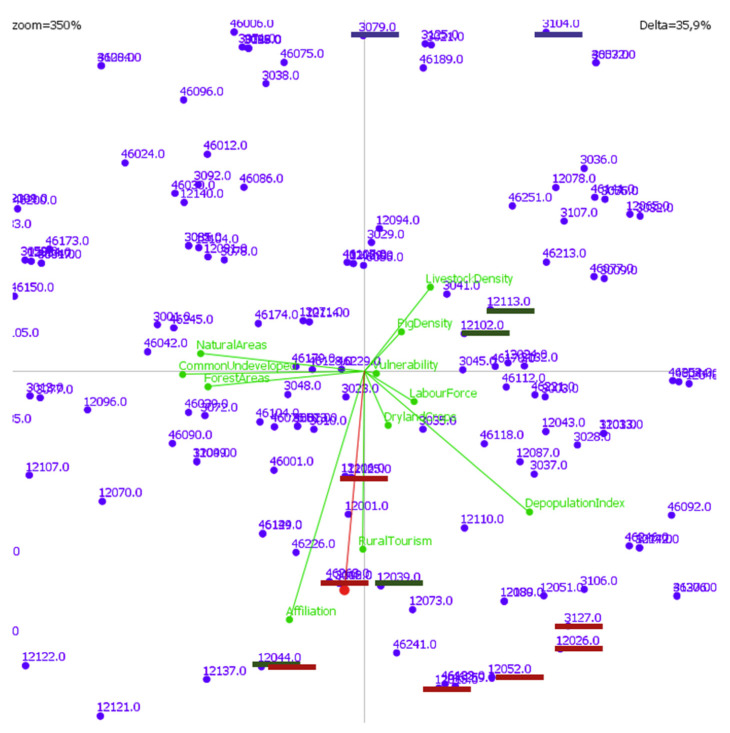
GAIA plane for municipalities with sustainable areas for pig farms.

**Figure 15:**
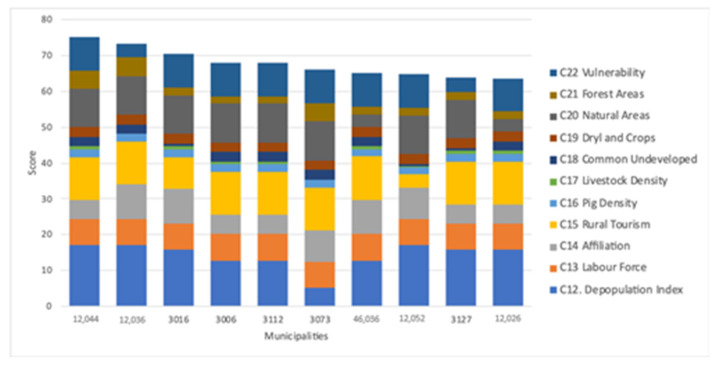
Contribution graph of the municipalities which are the best to establish new farms.

An important graphical tool to analyse multiple-criteria problems was shown in Figure 14, i.e., the GAIA plane. In this graph, the longer the axis of a criterion, the more discriminant is that criterion. As could be seen, the most discriminant criterion was social security affiliation evolution. The criteria with similar preferences had axes oriented approximately in the same direction, such as the percentage of protected natural areas and percentage of forest areas. On the other hand, the conflict criteria were oriented in opposite directions, for example, livestock farming density and rural tourism evolution. The criteria that were not linked to others in terms of preferences were characterised by orthogonal axes. If a municipality had a high score in one criterion, this area was in the direction of the axis of this criterion, such as municipality 12,039, which had the maximum score in the rural tourism evolution criterion. Finally, similar alternatives were shown by close points such as 12,102 and 12,113, which had the same score in all the criteria except in two of them. Examples of these areas are shown in Figure 15.

As shown in Figure 15, the contribution graph showed that the best municipalities to establish new farms were characterised by depopulation and low rural tourism, and they were located far from natural areas and with little vulnerability in groundwater. These four criteria were relevant for the evaluation of the feasible areas from the results of the AHP survey. There were municipalities with high scores, for example, 3073 and 12,052. The first differed in having a lower depopulation index but had fewer forest areas. On the contrary, the second had a higher rural tourism index compared to the rest that was represented in the figure. Municipalities 12,014 and 3035, even though they were among the 10 best, were removed because they did not have enough area to establish a new farm.

With the aim of testing the robustness of PROMETHEE results at the municipality with suitable livestock areas, a sensitivity analysis was carried out. The stability of the weights was analysed to maintain the ranking of the best municipalities. Only the reduction of two of the most important criteria, depopulation index and degree of vulnerability to groundwater pollution by a percentage of 8.16% and 26.33%, respectively, could make a change in the evaluation of the best municipalities. All of this highlighted the robustness of the analysis carried out and the selection of the best municipalities in which to establish livestock farms.

These results allowed the definition and ranking of suitable areas for the pig sector, using a wide range of socio-economic, sectoral, and environmental criteria. The main difference with the study presented here was the number and type of variables considered. The study we presented included a larger number of variables (22 variables) than studies analysing sustainable zones for livestock farming such as the study by [3,4,6], which considered six variables. In addition, socio-economic municipal variables that were not used in these studies were included. This larger number of variables allowed us to establish suitable areas more precisely, considering all regulatory aspects of the sector, and to remove unsuitable areas (STEPs 1 and 2); thus, only analysing those that were suitable in the next step. In a third step, the methodology applied selected the municipalities with the highest outranking according to socio-economic, sectoral, and environmental characteristics.

This procedure identified the suitable municipalities with scores presenting higher values and lower variability than those obtained in the studies of [1,21], using a multiple-criteria method. The hybrid approach using AHP and PROMETHEE was an outranking methodology that assessed the best municipalities in comparison with the rest. Thus, the model revealed which ones were the best to establish new pig farms. PROMETHEE as an outranking method was not compensatory and it was more discriminant than accumulative multi-criteria techniques [40].

In addition, the size of the farms was considered to determine the distances they should be separated from other farms or facilities such as slaughterhouses, landfills, and ABPs plants.

In summary, the results obtained indicated that the sector’s efforts should be focused on locating farms in municipalities according to their socio-economic characteristics and, secondly, by environmental factors. More specifically, areas suitable for the location of pig farms should be prioritised in municipalities at risk of depopulation or where rural tourism was decreasing. Secondly, suitable areas in municipalities with few natural protected areas and low vulnerability to groundwater should be prioritised.

## 4. Conclusions

The use of multi-criteria methods such as PROMETHEE and AHP, combined with GIS techniques, was used to assess the suitability of areas for pig farming development in a Mediterranean region with serious land-use conflicts. The study was carried out using 22 criteria reflecting sectoral, environmental, and urban planning regulatory factors and then socio-economic, sectoral, and environmental characteristics at the municipal level. The resulting map of municipalities with land suitable for the development of the swine sector was classified into four levels according to the score value (very high, high, moderate, low, and unsuitable). It was estimated that only 5% of the study area and 33% of the municipalities (178 municipalities) in the VC were suitable for livestock production. The characteristics that ruled out most of the area were due to urban planning regulations and recommendations on areas vulnerable to nitrates of agricultural origin and, to a lesser extent, to sectoral regulations. The municipalities with the best scores were selected mainly for their socio-economic characteristics and risk of depopulation. The outranking municipalities obtained demonstrated that the swine sector could be a strategic sector in half of the municipalities at risk of depopulation and in the municipalities where rural tourism did not increase as in the rest of the CV. This conclusion confirmed the initial hypothesis, highlighting livestock farming as a key sector for the revitalisation of rural areas at risk of depopulation, thus showing how administrations should design management instruments based on joint territorial and sectoral planning.

The results obtained made it possible to select suitable areas throughout the territory and allowed subsequent studies to analyse each of these areas to determine the size of the farms or farms that could be included in each of them.

In addition, this study opened the door to the analysis of livestock farming, distinguishing between intensive and extensive livestock species and their development possibilities depending on the characteristics of the territory. The methodology used could be applied in different areas and varying livestock species, for example, to study optimal and sustainable areas for extensive livestock species and their positive effects on population fixation in areas at risk of depopulation.

## Figures and Tables

**Table 1:** Number of pig farms and livestock units (LUs) in the Valencian Community (VC) by type.

	Alicante		Castellón		Valencia	
	Farms	LU	Farms	LU	Farms	LU
Reduced capacity	7	6.84	2	6.90	5	6.84
Group 1	7	255.08	228	17,117.30	136	11,021.11
Group 2	12	2978.30	302	63,198.29	159	33,074.28
Group 3	1	569.13	9	6657.05	16	10,798.64
Extended distance	6	808.28	16	1598.58	19	1756.18
Total	33	4617.63	557	88,578.12	335	56,657.05

**Table 2:** Source and format of the variables considered in STEPs 1, 2, and 3.

	Data Base	Source	Format
STEP 1	Municipal boundaries and study area	Spanish National Centre for Geographic Information	Shapefile
	Urban centres		
	Public roads		
	Pig farms	Georreferenced with a GPS	Database Table (Coordinates X,Y)
	Landfill sites	Integrated waste plan for the Valencia Region	Database Table (Coordinates X,Y)
	Slaughterhouses	List of approved establishments in the animal by-product field. Ministry of Agriculture, Fisheries, and Food. Spanish Government	Database Table (Coordinates X,Y)
	Meat industries		
	Animal by-products plants		
STEP 2	Nitrate-vulnerable zones	Decree 86/2018	Data base table (Municipalities with restriction)
	Non-developable land	Valencian Spatial Data Infrastructure	Shapefile
	Protected natural areas		
	Slope of the land		Obtained from a digital terrain model in raster format
STEP 3	Depopulation Index	Generalitat Valenciana’s statistical portal	Database table (Index municipalities)
	Labour force turnover rate		
	Social Security affiliations		
	Rural tourism evolution		
	Livestock farming	Georreferenced with a GPS	Database Table (Coordinates X,Y)
	Dryland crops	Spanish National Centre for Geographic Information	Shapefile
	Forest area	Valencian Spatial Data Infrastructure	Shapefile
	Vulnerability to groundwater pollution		

**Table 3:** Distances considered for criteria C_2_, C_3_, and C_6_.

	Criterion 2 (C_2_)			Criterion 3 (C_3_)	Criterion 6 (C_6_)
	**Group 1**	Groups 2–3	Extended Distance	Authorised Landfill	Animal By-Product Plants
Group 1	500 m	1 km	2 km	1 km	1 km
Groups 2 and 3	1 km	1 km	2 km	1 km	1 km
Extended distance	2 km	2 km	2 km	2 km	2 km

**Table 4:** Municipal depopulation indicators and thresholds.

Index	Description	Thresholds
Population density	Number of inhabitants per km^2^ in 2019	≤20
Demographic growth	Growth rate between 1999 and 2019 (%)	≤0%
Vegetative growth	Percentage representing the natural balance (difference between the number of births and deaths) in a given population (1999–2019)	≤−10%
Ageing rate	Ratio of the number of people aged 65 and over to the number of people aged 0–14 (1999–2019)	≥250%
Dependence index	Percentage of population under 15 and over 65 years of age	≥60%
Migratory rate	Migratory balance in 2009–2019 divided by total population in the last year (%)	≤0

Source: own creation based on the indicators proposed in Decree 182/2018.

**Table 5:** Evaluation table of alternatives.

Municipalities	Socio-Economics				Sectoral				Environmental		
	C_12_	C_13_	C_14_	C_15_	C_16_	C_17_	C_18_	C_19_	C_20_	C_21_	C_22_
A_1_	C_12_1_ (A_1_)	…	…	C_15_1_ (A_1_)	C_16_1_ (A_1_)	…	…	C_19_1_ (A_1_)	C_20_1_ (A_1_)	…	C_22_1_ (A_1_)
A_2_	C_12_2_ (A_2_)	…	…	C_15_2_ (A_2_)	C_16_2_ (A_2_)	…	…	C_19_2_ (A_2_)	C_20_2_ (A_2_)	…	C_22_2_ (A_2_)
…	…	…	…	…	…	…	…	…	…	…	…
A_i_	C_12_i_ (A_i_)	…	…	C_15_i_ (A_i_)	C_16_i_ (A_i_)	…	…	C_19_i_ (A_i_)	C_20_i_ (A_i_)	…	C_22_i_ (A_i_)
...	…	…	…	…	…	…	…	…	…	…	…
A_178_	C_12_178_ (A_178_)	…	…	C_15_178_ (A_178_)	C_16_178_ (A_178_)	…	…	C_19_178_ (A_178_)	C_20_178_ (A_178_)	…	C_22_178_ (A_178_)

**Table 6:** Evaluation table of alternatives.

Criteria	Preference Function	MAX/MIN
C_12_. Depopulation index	Usual	MAX
C_13_. Labour force turnover rate	Usual	MAX
C_14_. Evolution of social security	Usual	MAX
C_15_. Evolution or rural tourism	Usual	MAX
C_16_. Pig farming density	Usual	MAX
C_17_. Livestock farming density	Usual	MAX
C_18_. Percentage of available area of common undeveloped land	Usual	MAX
C_19_. Percentage of dryland crops	Usual	MAX
C_20_. Percentage of protected natural areas	Usual	MIN
C_21_. Percentage of forest areas	Usual	MIN
C_22_. Degree of vulnerability to groundwater pollution	Usual	MAX

**Table 7:** The aggregated weights from experts’ judgements by AHP for factors and criterion.

Factor	Criterion	Weights
Socio-economics		53.7%
	C_12_. Depopulation index	32.6%
	C_13_. Labour force turnover rate	23.5%
	C_14_. Evolution of social security	18.4%
	C_15_. Evolution or rural tourism	25.5%
Sectoral		13.6%
	C_16_. Pig farming density	28.7%
	C_17_. Livestock farming density	11.3%
	C_18_. Percentage of available area of common undeveloped land	25.9%
	C_19_. Percentage of dryland crops	34.2%
Environmental		32.7%
	C_20_. Percentage of protected natural areas	44.5%
	C_21_. Percentage of forest areas	18.0%
	C_22_. Degree of vulnerability to groundwater pollution	37.5%

**Table 8:** Classification criterion for evaluating municipalities with land suitable for the swine sector development.

Outranking Degree	Threshold		Municipalities (Number)	
	Lower	Upper	Number	%
Very highly outranked	57.39	75.19	43	24.16
Highly outranked	48.83	57.39	44	24.72
Moderately outranked	40.97	48.83	64	35.96
Low outranked	27.55	40.97	27	15.17
			178	100.00

## Data Availability

Not applicable.
